# Potassium fulvic acid alleviates salt stress of citrus by regulating rhizosphere microbial community, osmotic substances and enzyme activities

**DOI:** 10.3389/fpls.2023.1161469

**Published:** 2023-03-23

**Authors:** Manman Zhang, Xiaoya Li, Xiaoli Wang, Jipeng Feng, Shiping Zhu

**Affiliations:** ^1^ Citrus Research Institute, Southwest University, Chongqing, China; ^2^ National Citrus Engineering Research Center, Beibei, Chongqing, China

**Keywords:** potassium fulvic acid, microbial community, salt stress, citrus rootstock, distance-based redundancy analysis

## Abstract

Salt stress damage to plants has been becoming a global concern for agriculture. The application of potassium fulvic acid (PFA) is a promising strategy to alleviate the damage to plants and improve soil quality. However, the study of PFA on plant growth and rhizosphere microbial community remains limited. In this study, microcosmic experiments were conducted to verify the effect of PFA on citrus. Trifoliate orange (*Poncirus trifoliata*), the most important citrus rootstock, was used to evaluate the effect of PFA on salt damage. The results showed that PFA significantly increased the contents of chlorophyll a, chlorophyll b and carotenoid by 30.09%, 17.55% and 27.43%, and effectively avoided the yellowing and scorching of leaves under salt stress. Based on the results of two-way ANOVA, the mitigation of salt stress on trifoliate seedlings primarily attributed to the enhancement of protective enzyme activities, K^+^/Na^+^ ratio and the contents of soluble sugar, soluble protein and proline. Moreover, PFA enhanced neutral protease (S-NPT), sucrase (S-SC) and urease (S-UE) of rhizosphere soil and improved soil nutrition status. The abundance of *Bacillus*, a kind of rhizosphere beneficial bacteria, was improved by PFA under salt stress, which was mainly associated with the increased activities of S-NPT, S-SC and S-UE. Overall, the application of PFA showed great potential for the alleviation of salt damage on citrus.

## Introduction

Soil salinity, mainly caused by chaotic fertilization, poor irrigation and anthropogenic pollution, has seriously affected more than 6% of the world’s lands (Hassani et al., 2020; Mukhopadhyay et al., 2020). High salinity affects the growth and production of crops by preventing absorbing water and various nutrition elements from the soil (Mukhopadhyay et al., 2020; Sun et al., 2022; Zied et al., 2022). Citrus belongs to sweet-soil plant that is extremely sensitive to salt (Mokrani et al., 2020; Wei et al., 2020). Trifoliate orange (*Poncirus trifoliata*) is a widely used rootstock in citrus industry, which is sensitive to salt stress (Wei et al., 2021). Cl^-^ and Na^+^ are the major elements for citrus salt stress, and excessive Cl^-^ and Na^+^ can break the potential balance and increase the permeability of plasma membrane (Lin et al., 2015; Bankaji et al., 2019; Sun et al., 2022). Under salt stress, many adverse symptoms occur, such as chlorosis, scorching of leaf tip, early leaf falling and even trees dying (Wu et al., 2010; Navarro et al., 2014; Syvertsen and Garcia-Sanchez, 2014). Therefore, it is urgent to explore an effective, ecological and economic method to alleviate salt damage in citrus industry.

Fulvic acid (FA) is a short-chain molecular structure substance derived from natural humic acid, with high physiological activity and loading capacity (Islam et al., 2020; Brazien et al., 2021). FA can interact with oxides, hydroxides, metal ions, organic matter and minerals in the environment, effectively improve the availability of soil nutrients, reduce soil salinity, and promote plant growth (Brazien et al., 2021; Liu et al., 2022). Anjum et al. (2011) found that FA could enhance the activities of various antioxidant enzymes and protect plants from oxidative damage. The pre-treatment of FA improved salt tolerance of soybean (*Glycine max* L.) by maintaining ion balance (Dinler et al, 2016). In addition, Elrys et al. (2020) reported that under salt stress FA could promote the growth of wheat by reducing the level of active oxygen species and improving the antioxidant defense system. Potassium fulvic acid (PFA) is formed with FA by chelating K^+^ and rich in exchangeable K (Qiao et al., 2022). Compared with FA, PFA has superior advantages in composition, performance, price and physiological activity (He et al., 2022; Qiao et al., 2022; Wang et al., 2022). Although PFA is widely used in improving soil properties and crop production, it is rarely used as a salt hazard mitigation agent in plant under salt stress.

Soil microorganism directly or indirectly interacts with the roots of plant to influence the growth and health (Berendsen et al., 2012; Bai et al., 2015). Simultaneously, roots affect the functions and structures of soil microbial community through the root exudates and the metabolic activities of the marginal cells (Bai et al., 2015; Hartmann et al., 2015; Fu et al., 2017). Recently, it has been proved that the interaction between soil microorganisms and plant roots mainly depends on the rhizosphere microorganisms, which play key roles in response to biotic and abiotic stresses (Qu et al., 2020; Liu et al., 2021). For example, rhizosphere beneficial bacteria (i.e., *Pseudomonas*, *Rhizobium*, *Bacillus*, *Pantoea*, *Berkholderia*, *Microbacterium*, *Achromobacter* and *Methylbacillus*) significantly improved the tolerance of hosts to salt stress (Grover et al., 2011). Rhizosphere growth-promoting bacteria (PGPR) can alleviate the damage of salt stress on plants through selective absorption of Na^+^, K^+^ and Ca^2+^, and maintaining a high K^+^/Na^+^ ratio (Rojas-Tapias et al., 2012; Han et al., 2014). Ashraf et al. (2004) reported that the extracellular polysaccharide secreted by PGPR strains can not only bind cations, but also promote the formation of biofilm on the root surface, thus limiting the inflow of Na^+^ into the body of plants. Consequently, the response of plant rhizosphere microbial community to the alleviation of salt damage on citrus should be considered.

In this study, the effect of PFA on the growth of trifoliate seedlings under salt stress was investigated with microcosmic experiments. We hypothesized that PFA could alleviate salt damage on citrus by regulating rhizosphere microbial community and enhancing the physicochemical properties and nutrient status of soil. The main objectives of this study were to: (i) investigate the effect of PFA on the growth and the characteristics of biochemistry and physiology of citrus under salt stress; (ii) evaluate the effect of PFA on the physicochemical properties and nutrient status of rhizosphere soil; (iii) illustrate the effect of PFA on microbial community of citrus root and rhizosphere soil; and (i) explore the correlations between properties, enzyme activities of rhizosphere soil and dominant microorganisms. We expected that the new insights from our study would contribute to further application of PFA for the alleviation of salt damage on citrus.

## Material and methods

### Microcosmic experiment design and sample collection

The mineral PFA was purchased from Stanley Agricultural Group Co., Ltd (Linyi City, Shandong Province, China). The soil sample (0-20 cm in depth) was collected from a citrus orchard located in Chongqing City, China (29.76^°C^N, 106.38^°C^E). The soil samples were air-dried, ground and screened with a sieve (aperture of 2 mm). The visibly similar citrus rootstock trifoliate orange (*Poncirus trifoliata*) seedlings (5 days after germination) grown under the same conditions were transferred to a plastic pot filled with the soil (250 g, with one seedling in each pot). The seedlings were treated as follows 30 days after transplanting: treated firstly with PFA solution (100 mg/L, 20 mL) for three times at an interval of one week, and then with NaCl solution (100 mM, 20 mL) for three times at an interval of one week (PFA+SS); treated with PFA solution as mentioned above, and then with equal amount of water (PFA); treated with equal amount of water in the first three weeks, and then with NaCl solution (100 mM, 20 mL) (SS); treated with only water during the whole process (CK). Each treatment contained a total of 45 seedlings and they were used for three biological repeats. The experiments were conducted in a greenhouse with temperature (25 ± 2°C) and humidity (50%-60%). The rhizosphere soil, fresh roots and leaves of the seedlings were collected 7 days after treatment.

### Growth and characteristics analysis of citrus

The plant height, root length, and fresh/dry biomass of the seedlings were measured. The contents of chlorophyll a, chlorophyll b and carotenoid were determined by using spectrophotometry (Shang et al., 2021). The root vigor and the contents of soluble protein, soluble sugar, proline and malondialdehyde (MDA) in fresh roots and leaves of the seedlings were analyzed using the corresponding test kits (Nanjing Boyan Biotechnology Co., Ltd., Nanjing City, Jiangsu Province, China). In addition, the activities of peroxidase (POD), catalase (CAT) and superoxide dismutase (SOD) of the seedling roots and leaves were measured using different enzyme-linked immunosorbent assay (ELISA) kits (Nanjing Boyan Biotechnology Co., Ltd., Nanjing City, Jiangsu Province, China), respectively. Based on the digestion systems of concentrated HNO_3_ and HClO_4_ (4:1, v/v), the concentrations of Na^+^ and K^+^ in the roots and leaves of the seedlings were determined with a flame atomic absorption spectrophotometer (Sun et al., 2022).

### Physicochemical properties and enzyme activities analysis of the rhizosphere soil

The values of electrical conductivity (EC) and pH of the rhizosphere soil samples were all determined at a water/soil mass ratio of 5:1. The contents of ammonium nitrogen (AN) and nitrate nitrogen (NN) in rhizosphere soil samples were detected using Skalar Continuous Flow Analyzer 5000 (Skalar Analytical BV, Breda, Netherlands). The available phosphorus (AP) contents of soil samples were estimated using the ammonium acetate-flame photometer method (Yin et al., 2022). In addition, the potassium dichromate heating oxidation-volumetric method was used for the determination of organic matter (OM). The activities of the soil catalase (S-CAT), neutral protease (S-NPT), sucrase (S-SC) and urease (S-UE) were determined using enzyme activity test kits (Nanjing Boyan Biotechnology Co., Ltd., Nanjing City, Jiangsu Province, China), according to the manufacturer’s instructions.

### DNA extraction, PCR amplification, and 16S rRNA gene amplicon sequencing of root and rhizosphere soil samples

The FastDNA Spin Kit for Soil (MP Biomedicals, CA, United States) was used to extract the genomic DNA from the entire roots and rhizosphere soils. Following the manufacturer’s instructions, 50 μL of DNA was extracted from root (250 mg) and rhizosphere soil (300 mg), and then stored at -20°C for further analysis. The universal primers 799F/1193R and ITS1F/ITS2R were used to amplified the V5-V7 regions of the 16S rRNA genes and ITS region 1 of the nuclear ribosomal coding cistron of the extracted DNA, respectively. The PCR amplification were performed according to Zheng et al. (2021) and Shang et al. (2021). After PCR amplification, the bands were excised and purified with 2% agarose gel using a PCR Purification Kit. The purified PCR products from all samples were collected, and paired-end sequencing was performed on the Illumina MiSeq sequencing platform (Shanghai Majorbio Bio-pharm Technology Co., Ltd., Shanghai City, China).

### Amplicon sequence processing and bioinformatics analysis

Restricting the data to similarity of ≥97%, operational taxonomic unit (OTU) clustering of non-repetitive sequences was obtained using UPARSE (version 7.0.1090). The *α*-diversity (Chao 1 index) was calculated using *Mothur* software. To visualize the variations in microbial compositions, the principal components analysis (PCoA) of the treatments were applied using *QIIME* and *R* software (v. 3.5.3). The distance-based redundancy analysis (db-RDA) screened the environmental factors on the Majorbio i-Sanger Cloud Platform (Shanghai Majorbio Bio-pharm Technology Co., Ltd., Shanghai City, China, https://cloud.majorbio.com). Sequence data have been deposited in the NCBI Sequence Read Archive under BioProject number PRJNA935538.

### Statistical analysis

Statistical analysis and figure creations was conducted using *SPSS Statistics 20.0* (Chicago, USA) and *Origin 8.5* (Northampton, USA), respectively. The significances between two different treatments were analyzed by Student’s t test. Two-way ANOVA analyses were conducted to analyze the significance of interactions between PFA and SS. Data in the tables are expressed as means ± standard error (n = 3).

## Results

### Effect of PFA on the growth and root vigor of citrus seedlings

**Figure 1:**
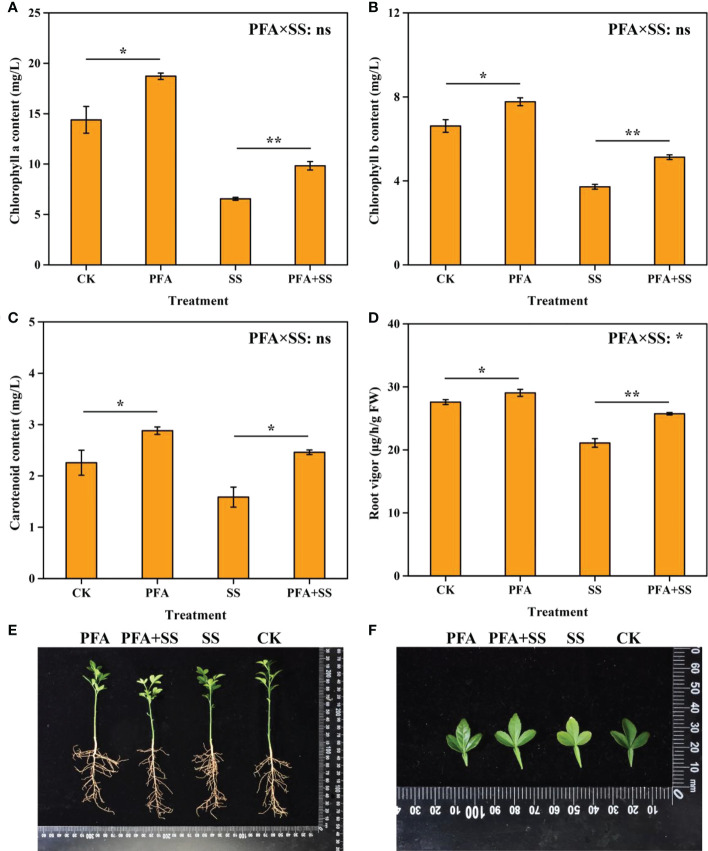
The contents of chlorophyll a **(A)**, chlorophyll b **(B)**, carotenoid **(C)** and root vigor **(D)**, plant growth **(E)**, leaves **(F)** of the seedlings. CK, the seedlings without PFA and salt stress; PFA, the seedlings amended with PFA and without salt stress; SS, the seedlings with salt stress; PFA+SS, the seedlings amended with both PFA and salt stress. PFA × SS, interaction between PFA and SS. The error bars represent the standard error (n = 3). “ns”, “*”, and “**” mean not significant, *p* < 0.05 and *p* < 0.01 according to Student’s t test and two-way ANOVA, respectively.

The plant height, root length, fresh biomass and dry biomass of the seedlings are shown in **Figure S1**. Although PFA had no significant effect on plant height, root length, fresh biomass, dry biomass of the seedlings (**Figure S1**), PFA significantly increased the contents of chlorophyll a, chlorophyll b and carotenoid by 30.09%, 17.55% and 27.43%, respectively (**Figures 1A–C**). In addition, PFA+SS significantly elevated the chlorophyll a, chlorophyll b and carotenoid of leaves and the root vigor, compared to SS treatment (**Figures 1A–D**). Two-way ANOVA revealed a significant positive interaction of PFA × SS treatment on root vigor of the seedlings (**Figure 1D**), indicating that PFA had a greater promoting effect on root vigor under salt stress, which was conducive to the root growth under salt stress. Under salt stress, the leaves of trifoliate seedlings gradually appeared yellow and scorched, but PFA+SS significantly alleviated the harm of salt stress to the seedlings (**Figures 1E, F**).

### Effect of PFA on the biochemical and physiological characteristics of the seedlings

#### Protective enzyme activities

**Figure 2:**
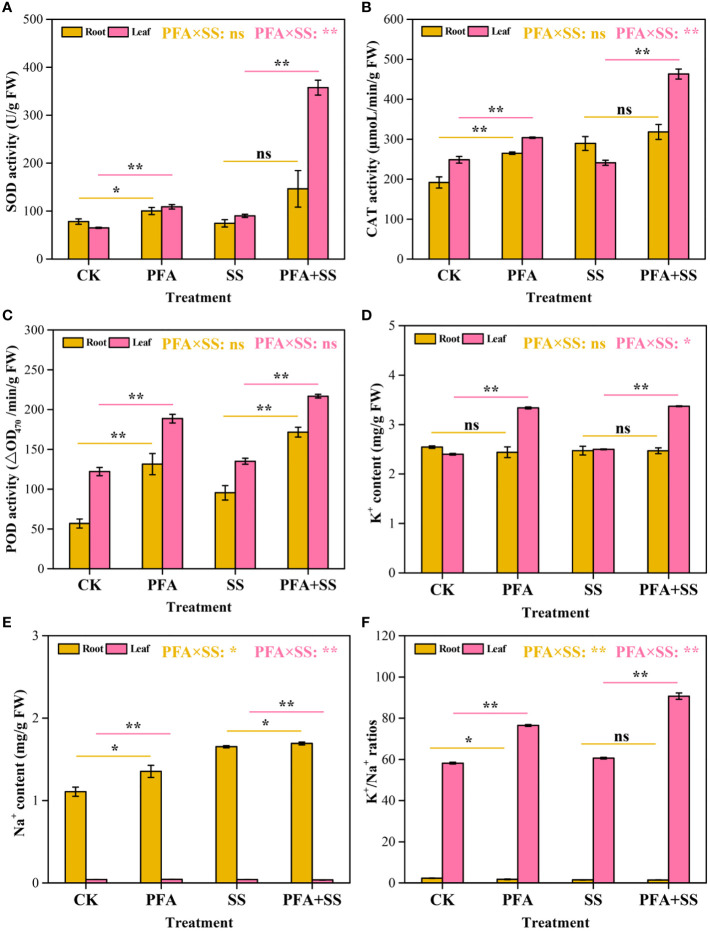
The activities of SOD **(A)**, CAT **(B)**, POD **(C)** and K^+^ content **(D)**, Na^+^ content **(E)**, K^+^/Na^+^ ratios **(F)** in the seedlings. CK, the seedlings without PFA and salt stress; PFA, the seedlings amended with PFA and without salt stress; SS, the seedlings with salt stress; PFA+SS, the seedlings amended with both PFA and salt stress. PFA × SS, interaction between PFA and SS. The error bars represent the standard error (n = 3). “ns”, “*”, and “**” mean not significant, *p* < 0.05 and *p* < 0.01 according to Student’s t test and two-way ANOVA, respectively.

The activities of protective enzymes in the seedlings were affected by PFA (**Figures 2A–C**). PFA and PFA+SS treatments enhanced the activities of SOD and CAT in the seedling leaves compared to CK and SS treatments, respectively (**Figures 2A, B**). Two-way ANOVA revealed highly significant positive effects of PFA × SS treatment interaction on the activities of SOD and CAT in leaves, showing the greater promoting effects of PFA under salt stress, which means that PFA can effectively alleviate the damage of citrus caused by SS. Moreover, the activities of POD in root and leaf samples were significantly increased with the addition of PFA under salt stress or without salt stress (**Figure 2C**). However, the results of two-way ANOVA demonstrated that there was no significant PFA × SS treatment interaction on the POD activities.

#### Accumulation of K^+^ and Na^+^

The contents of K^+^ and Na^+^ in the roots and leaves are displayed in **Figure 2**. PFA and PFA+SS treatments generally increased the contents of K^+^ in the leaves, but there was no significant difference in the root samples (**Figure 2D**). SS and PFA+SS treatments enhanced the accumulation of Na^+^ in the roots, and PFA+SS conspicuously decreased Na^+^ content in the leaves, compared to SS treatment (**Figure 2E**). Two-way ANOVA revealed a significant negative effect of PFA × SS treatment interaction on the Na^+^ contents in the leaves of the seedlings, which showed that PFA reduced the accumulation of Na^+^ under salt stress. And decreased content of Na^+^ mitigated the harmful effects of salt on the seedlings. In root samples, the K^+^/Na^+^ ratios were dramatically decreased with the treatments of PFA, compared to CK treatment. However, PFA and PFA+SS treatments increased the K^+^/Na^+^ ratios in the leaves by 31.46% and 49.72%, compared to CK and SS treatments, respectively (**Figure 2F**). Especially, PFA × SS treatment interaction showed a significant positive effect on the K^+^/Na^+^ ratio in the leaves, which was beneficial to alleviate salt stress on citrus.

#### Soluble sugar, soluble protein, proline and MDA contents

**Figure 3:**
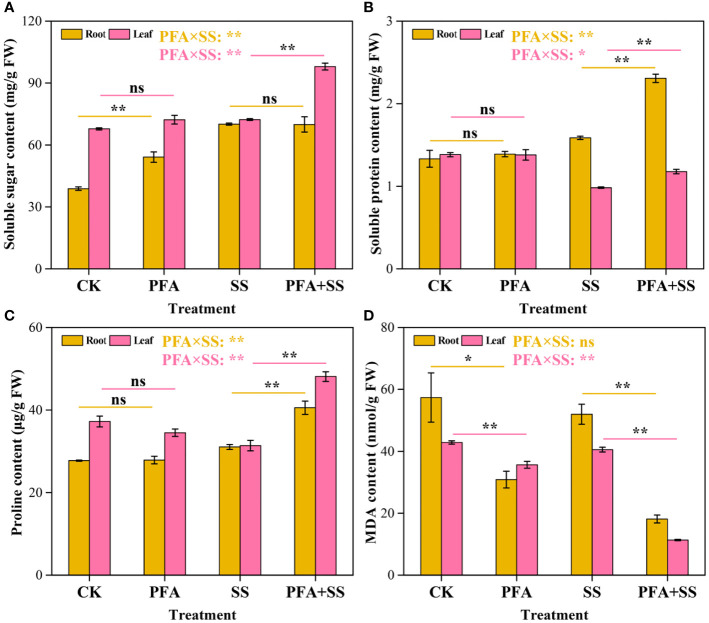
The contents of soluble sugar **(A)**, soluble protein **(B)**, proline **(C)** and MDA **(D)** in the seedlings. CK, the seedlings without PFA and salt stress; PFA, the seedlings amended with PFA and without salt stress; SS, the seedlings with salt stress; PFA+SS, the seedlings amended with both PFA and salt stress. PFA × SS, interaction between PFA and SS. The error bars represent the standard error (n = 3). “ns”, “*”, and “**” mean not significant, *p* < 0.05 and *p* < 0.01 according to Student’s t test and two-way ANOVA, respectively.

PFA affected the contents of osmoregulation substances (**Figure 3**). PFA treatment significantly increased soluble sugar contents in the roots by 39.34% compared to CK treatment, but there was no significant effect of PFA on the contents in root samples under salt stress (**Figure 3A**). In addition, the contents of soluble sugar in the leaves exhibited an extremely significant increase under PFA+SS treatment. The soluble protein contents were increased by 45.28% and 20.41% in the roots and leaves with PFA+SS treatment, compared to SS treatment (**Figure 3B**). However, PFA treatment showed no significant improvement effect on the soluble protein contents of the seedlings. For the proline contents, there were no significant differences between CK and PFA treatments in root and leaf samples, but PFA+SS treatment significantly promoted the synthesis of proline in the seedlings compared to SS treatment (**Figure 3C**). Moreover, two-way ANOVA indicated that PFA × SS treatment interaction had highly significant positive influences on the soluble sugar, soluble protein and proline contents in leaves, which were beneficial to the growth of plants, indicating that PFA had greater promoting effects of them under salt stress. Compared to CK treatment, the contents of MDA in the root and leave samples decreased under PFA treatments (**Figure 3D**). Notably, PFA+SS treatment significantly decreased MDA contents in the roots and leaves by 65.10% and 71.96%, compared to SS treatment, respectively (**Figure 3D**). Two-way ANOVA also showed that PFA × SS treatment interaction had a highly significant negative influence on the content of MDA in leaves, of which excessive accumulation was harmful to plants, indicating that PFA has a better inhibition effect on MDA under stress.

### Effect of PFA on the physicochemical properties and enzyme activities of rhizosphere soil

The effect of PFA on rhizosphere soil physicochemical properties is provided in **Table 1**. PFA showed no significant effect on the pH values of the soil samples, but there were significant effects of PFA and PFA+SS treatments on the soil EC values. PFA treatment significantly decreased the soil EC value by 47.78%, compared to CK, and the results of two-way ANOVA also demonstrated a highly significant PFA × SS treatment interaction on the EC values of soil. Furthermore, PFA treatment significantly promoted soil OM contents by 37.75%. Specifically, PFA amendment significantly improved soil AN content from 5.05 mg/kg to 6.73 mg/kg, but significantly decreased soil NN content by 38.46%. The contents of OM and AN were not significantly affected by PFA under salt stress, but PFA+SS treatment significantly increased the NN contents compared to SS treatment. As for the effect on AP content of rhizosphere soil, PFA+SS treatment showed no significant effects on soil AP content compared to SS treatment, but PFA treatment significantly increased soil AP content by 231.06%. Two-way ANOVA revealed a highly significant positive effect of PFA× SS treatment interaction on the NN contents of soil, suggesting that PFA had greater promoting effects on them under stress.

**Table 1:** Effect of PFA on the rhizosphere soil properties of the seedlings.

Soil properties	Treatment				Significance
	CK	PFA^a^	SS	PFA+SS^b^	PFA×SS^c^
pH	8.22 ± 0.01	8.20 ± 0.00 ns	8.13 ± 0.05	8.08 ± 0.02 ns	ns
EC (μS/cm)	642.5 ± 15.08	335.5 ± 18.21**	675.25 ± 10.05	617.75 ± 8.18**	**
OM (g.kg^-1^)	6.49 ± 0.08	8.94 ± 0.53**	8.11 ± 0.90	10.69 ± 1.05ns	ns
AN (mg.kg^-1^)	5.05 ± 0.21	6.73 ± 0.26**	4.95 ± 0.28	4.97 ± 0.19ns	**
NN (mg.kg^-1^)	0.39 ± 0.01	0.24 ± 0.01**	0.36 ± 0.01	0.57 ± 0.01**	**
AP (mg.kg^-1^)	2.35 ± 0.09	7.78 ± 0.54**	6.18 ± 0.23	6.71 ± 0.34ns	**

EC, Electrical conductivity; OM, Organic matter; AN, Ammonium nitrogen; NN, Nitrate nitrogen and AP, Available phosphorus. CK, the seedlings without PFA and salt stress; PFA, the seedlings amended with PFA and without salt stress; SS, the seedlings with salt stress; PFA+SS, the seedlings amended with both PFA and salt stress. PFA × SS, interaction between PFA and SS. ^a^ “ns” and “**” mean not significant, and p < 0.01 between CK and PFA treatments according to Student’s t test; ^b^ “ns” and “**” mean not significant, and p < 0.01 between SS and PFA+SS treatments according to Student’s t test; ^c^ “ns” and “**” mean not significant interaction of PFA × SS treatment, and p < 0.01 according to two-way ANOVA.

The effect of PFA on the enzymatic activities of the citrus rhizosphere soil is presented in **Table 2**. There was no significant effect of PFA treatment on S-CAT activity, but PFA+SS treatment remarkably increased S-CAT by 28.74%, compared to SS treatment. In addition, SS treatment decreased S-NPT activity from 41.30 μg·h^-1^·g^-1^ to 26.16 μg·h^-1^·g^-1^, but PFA and PFA+SS treatments dramatically promoted S-NPT activity by 23.22% and 159.10%, compared to CK and SS treatments, respectively. Similarly, the results of two-way ANOVA showed that PFA × SS treatment interaction had a highly significant positive effect on the activities of S-NPT, indicating a better promotion of PFA on S-NPT activities under salt stress. The activities of S-SC were dramatically higher by 69.95% under PFA amendment. In addition, the significant differences of S-UE activities were observed between CK and PFA treatments, SS and PFA+SS treatments. The significant PFA × SS treatment interaction also affected the activities of S-SC and S-UE positively.

**Table 2:** Effect of PFA on the rhizosphere soil enzyme activities of the seedlings.

Soil enzyme activities	Treatment				Significance
	CK	PFA^a^	SS	PFA+SS^b^	PFA×SS^c^
S-CAT (μmol·h^-1^·g^-1^)	228.70 ± 13.00	231.08 ± 16.14ns	164.31 ± 6.88	211.54 ± 19.53**	*
S-NPT (μg·h^-1^·g^-1^)	41.30 ± 2.70	50.89 ± 2.12**	26.16 ± 4.40	67.78 ± 6.41**	**
S-SC (mg·d^-1^·g^-1^)	2.13 ± 0.38	3.62 ± 0.41**	2.15 ± 0.26	5.50 ± 0.16**	**
S-UE (μg·d^-1^·g^-1^)	42.92 ± 2.33	47.22 ± 1.32*	36.69 ± 2.29	63.11 ± 8.59**	**

S-CAT: soil catalase, S-NPT: soil neutral protease, S-SC: soil sucrase, S-UE: soil urease. CK: the seedlings without PFA and salt stress; PFA: the seedlings amended with PFA and without salt stress; SS: the seedlings with salt stress; PFA+SS: the seedlings amended with both PFA and salt stress. PFA × SS: interaction between PFA and SS. ^a^ “ns”, “*”, and “**” mean not significant, p < 0.05 and p < 0.01 between CK and PFA treatments according to Student’s t test; ^b^ “ns”, “*”, and “**” mean not significant, p < 0.05 and p < 0.01 between SS and PFA+SS treatments according to Student’s t test; ^c^ “ns”, “*”, and “**” mean not significant interaction of PFA × SS treatment, p < 0.05 and p < 0.01 according to two-way ANOVA.

### Effect of PFA on the microbial diversity and community of root and rhizosphere soil

**Figure 4:**
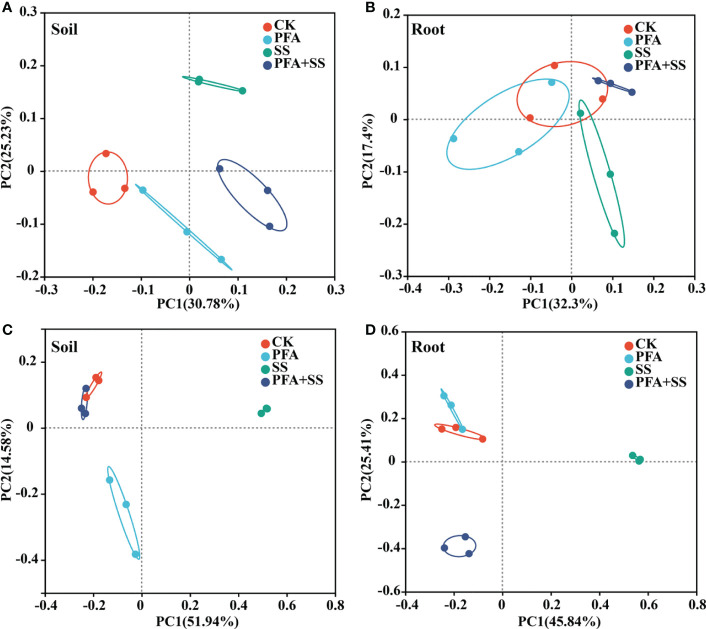
Principal components analysis (PCoA) based on the microbial community of the seedlings at the OTU level. **(A)** Bacterial community for the rhizosphere soil sample; **(B)** bacterial community for the root sample; **(C)** fungal community for the rhizosphere soil sample; **(D)** fungal community for the root sample. CK, the seedlings without PFA and salt stress; PFA, the seedlings amended with PFA and without salt stress; SS, the seedlings with salt stress; PFA+SS, the seedlings amended with both PFA and salt stress.

Based on the 16S rRNA gene and ITS amplicon sequencing, a total of 223,572 and 813,072 clean reads were obtained, respectively. The Venn diagram revealed that 452 and 172 bacterial OTUs, 238 and 73 fungal OTUs were shared among the all treatments in the roots and rhizosphere soils (**Figure S2**). In addition, 48–184, 69–104, 62–121 and 31–98 unique microbial OTUs were observed for CK, FPA, SS and FPA+SS treatments, respectively. PFA treatment significantly decreased the bacterial Chao1 index in the rhizosphere soil samples (**Figure S3A**), but the PFA and salt stress showed no significant effects on the fungal Chao1 indexes of rhizosphere soil and root samples of trifoliate seedling (**Figures S3C, D**). PCoA on the OTU level showed that the soil bacterial and fungal communities in the different treatments were entirely distributed in different quadrants (**Figures 4A, C**). However, the bacterial communities of root samples in the CK were similar to that in the PFA, SS, and PFA+SS treatments (**Figure 4B**). The PC1 and PC2 axis totally explained 25.41%–45.84% of the variation (**Figure 4D**), indicating the shifts in the composition of root fungal communities under different treatments.

**Figure 5:**
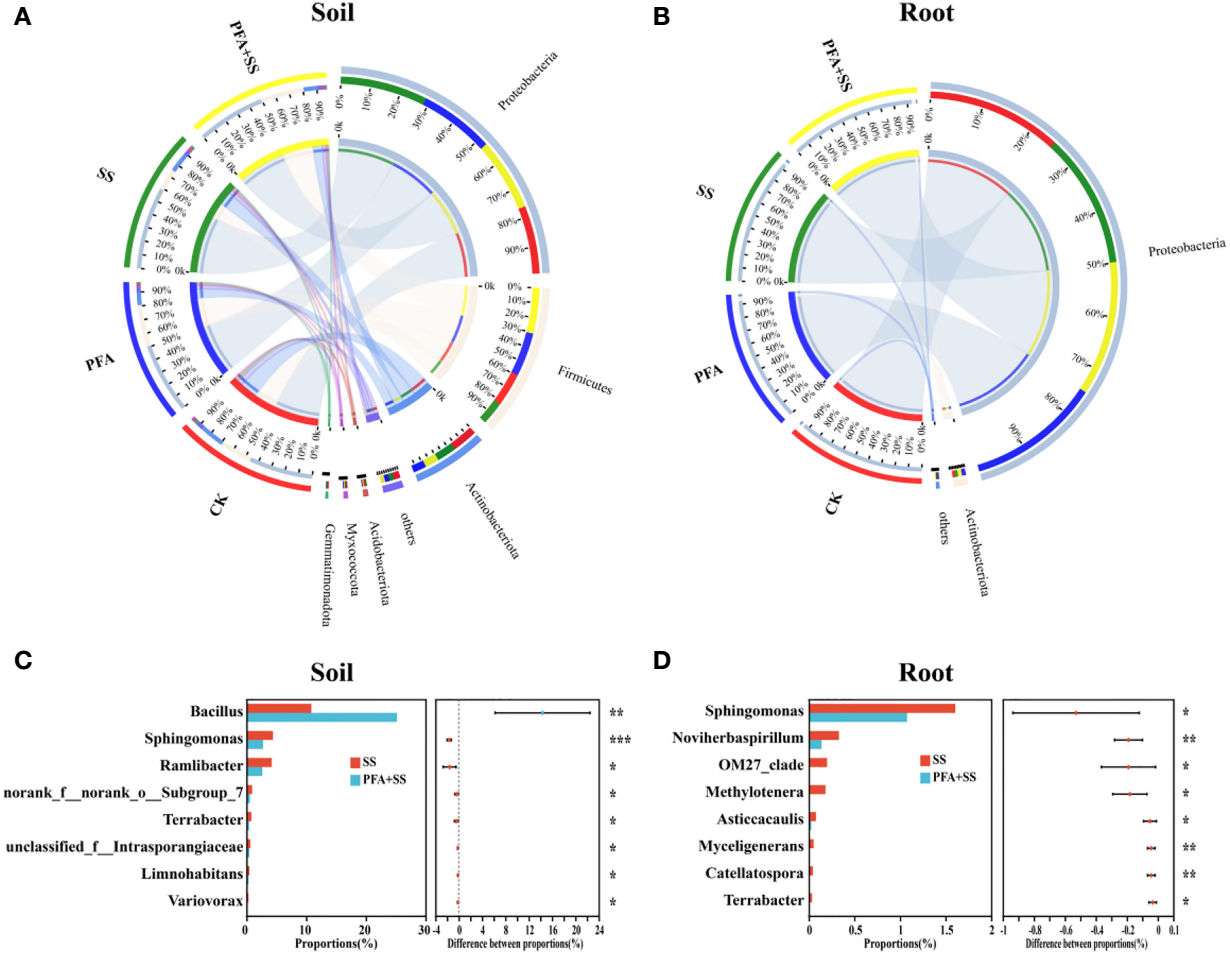
The relative abundance and composition of bacterial communities in the rhizosphere soil **(A)** and root **(B)** samples at the phylum level, the significance comparisons of bacterial communities in the rhizosphere soil **(C)** and root **(D)** samples at the genus level. CK, the seedlings without PFA and salt stress; PFA, the seedlings amended with PFA and without salt stress; SS, the seedlings with salt stress; PFA+SS, the seedlings amended with both PFA and salt stress. *p<0.05, **p<0.01, and ***p<0.001.

The microbiota of root and rhizosphere soil samples at the phylum level are shown in **Figures 5A, B**, **S4A, B**. For the rhizosphere soil samples, the dominant bacterial phyla (relative abundance >10%) in all treatments were Proteobacteria (47.36%–60.75%), Firmicutes (16.25%–32.32%) and Actinobacteria (9.50%–18.40%) (**Figure 5A**), while only Proteobacteria (>94.67%) was the most dominant in root samples (**Figure 5B**). The predominant fungal phylum of rhizosphere soil and root samples were Basidiomycota (27.26%–74.89%) and Ascomycota (14.86%–46.67%) in all treatments (CK, PFA, SS and PFA+SS) (**Figures S4A, B**). Moreover, the comparative response of bacterial and fungal communities between SS and PFA+SS in the roots and rhizosphere soils was observed at genus level (**Figures 5C, D**, **S4C, D**). Comparing with SS, PFA+SS treatment significantly enhanced the relative abundances of *Bacillus* and decreased *Sphingomonas* abundances (**Figures 5C, D**). The PFA and salt stress treatments significantly influenced a total of 7 fungal genera abundances in the rhizosphere soil and root samples, in which the two most dominant genera (*Rhizoctonia* and *Ceratobasidium*) were increased by PFA+SS (**Figures S4C, D**).

### Correlations between rhizosphere soil properties, enzyme activities and dominant microorganisms of the seedlings

**Figure 6:**
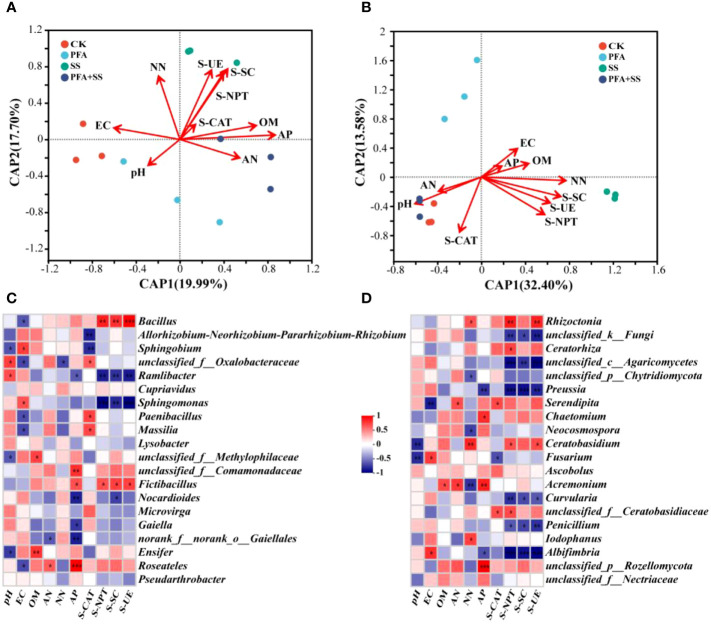
The db-RDA of bacterial **(A)**, fungal **(B)** communities and properties, enzyme activities in the rhizosphere soil of the seedlings, and correlation heat maps of properties, enzyme activities and the relative abundances of top 20 dominant genera of bacteria **(C)**, fungi **(D)** in the rhizosphere soil samples. CK, the seedlings without PFA and salt stress; PFA, the seedlings amended with PFA and without salt stress; SS, the seedlings with salt stress; PFA+SS, the seedlings amended with both PFA and salt stress. EC, Electrical conductivity; OM, Organic matter; AN, Ammonium nitrogen; NN, Nitrate nitrogen and AP, Available phosphorus. S-CAT, soil catalase; S-NPT, soil neutral protease; S-SC, soil sucrase; S-UE, soil urease. *p<0.05, **p<0.01, and ***p<0.001.

The db-RDA was carried out to evaluate the correlations between properties, enzyme activities of soil and dominant microbial communities (**Figures 6A, B**). Of the soil properties indicators, soil AP content was mostly affected by the soil bacterial communities, followed by OM, NN and AN (**Figure 6A**). In addition, soil AN content was negatively with NN content. As for the enzyme activities of rhizosphere soil samples, S-NPT was positively correlated with S-SC, S-UE and S-CAT. Especially, SS treatment had the most significant effect on the S-NPT, S-SC and S-UE activities. The soil EC value was significantly correlated with the relative abundances of 7 bacterial genera and 3 fungal genera, respectively (**Figures 6C, D**). *Bacillus* and *Fictibacillus* abundances were significantly positively correlated with S-NPT, S-SC and S-UE (**Figure 6C**). Moreover, AP was significantly correlated with 4 fungal genera, including *Preussia*, *Chaetomium*, *Acremonium* and *Albifimbria* (**Figure 6D**). *Fusarium* had significantly positive correlation with soil EC, but negative with soil pH and S-CAT. The relative abundances of *Cupriavidus*, *Lysobacter*, *Microvirga*, *Pseudarthrobacter*, *Ascobolus* had no significant correlation with physicochemical properties and enzyme activities of rhizosphere soil samples.

## Discussion

Recently, large numbers of evidences have showed that PFA serves as a promising strategy to alleviate the damage to plants caused by various abiotic stresses (Elrys et al., 2020; Qiao et al., 2022; Wang et al., 2022). In this study, we found that PFA avoided yellowing and scorching of leaves and significantly increased the contents of chlorophyll a, chlorophyll b and carotenoid of the seedlings (**Figure 1**), but PFA × SS treatment interaction showed no significant effect, indicating that PFA can effectively promote the growth status of citrus with or without salt stress.

The protective enzyme systems of plant play important roles in response to environmental stress (Abdelaal et al., 2019; Unoki et al., 2020). As important members of the plant protective enzyme systems, the activities of CAT, SOD and POD were used to evaluate the resistance of citrus to salt stress in this study. Excessive accumulation of O_2_^-^ in plants can provoke oxidative stress, which cause toxicity and show symptoms (Shang et al., 2021). However, SOD and POD of plants can convert O_2_^-^ into H_2_O_2_, and then, CAT effectively remove H_2_O_2_ to reduce oxidative damage to the cell membrane systems (Abdelaal et al., 2019; Che et al., 2020; Shang et al., 2021). In this study, PFA+SS treatment significantly enhanced the activities of CAT, SOD and POD in the leaves of citrus compared to SS treatment, and two-way ANOVA revealed highly significant positive effects of PFA × SS treatment interaction on the activities of SOD and CAT in leaves (**Figures 2A–C**), indicating that PFA had greater promoting effects of them and alleviated the oxidative damage caused by saline stress *via* the protective enzyme system (**Figures 1E, F**). Additionally, the accumulation of reactive oxygen species (ROS) in plants dramatically increased the MDA content caused by membrane lipid peroxidation (Li et al., 2017; Sun et al., 2022). The increased contents of osmoregulation substances, such as soluble sugar, soluble protein and proline, effectively removed ROS, reducing the content of MDA in plants, and thus relieved the damage of salt stress to plants (Wang et al., 2013; Dien et al., 2019; Kucerova et al., 2019; Lin et al., 2019; Gurrieri et al., 2020; Sun et al., 2022). The relief of symptoms in leaves induced by PFA (**Figures 1E, F**) might be related to the highly significant positive PFA × SS treatment interactions on the contents of soluble sugar, soluble protein and proline (**Figures 3A–C**) and the highly significant negative PFA × SS treatment interaction on MDA contents (**Figure 3D**), which caused the significantly increased contents of soluble sugar, soluble protein, proline and the decreased content of MDA under salt stress.

Properties and nutrient contents of soil directly affect plant growth and productivity (Ma et al., 2021). The high EC value of soil reflects the over-concentration of soluble salt, which is not conducive to plant growth (Sun et al., 2022; Yin et al., 2022). In this study, PFA treatment significantly reduced the EC value of salt-stressed soil (**Table 1**), indicating the alleviation of salt damage under PFA+SS treatment. Furthermore, PFA directly provides nutrients and also improves soil nutrient availability for the plants. In this study, PFA improved physicochemical properties of the soil under salt stress, such as OM, NN and AP (**Table 1**), which is in agreement with the report of Kumar Sootahar et al. (2019) and Sun et al. (2022).

Soil enzyme is an important driving force of soil nutrient metabolism, and its activity can directly reflect the intensity and direction of soil nutrient conversion and the biochemical process of soil (Fei et al., 2019; Aponte et al., 2020). S-CAT, an important indicator of soil micro-ecological environment, is related to soil respiration intensity and soil microbial activity, which can effectively prevent the toxicity of hydrogen peroxide (Aponte et al., 2020; Shang et al., 2021). In this study, PFA+SS treatment significantly enhanced the activity of S-CAT compared to SS treatment (**Table 2**), which was consistent with the report by Zhu et al. (2021). Additionally, S-UE can promote the hydrolysis of urea to ammonia and the supply of nitrogen nutrition for plants (Lee et al., 2021). S-SC can catalyze the hydrolysis of oligosaccharides in soil to glucose, fructose and other monosaccharides available to plants, and participate in the soil organic carbon cycle (Farooq et al., 2021). S-NPT is mainly involved in the conversion of amino acids, proteins and other organic compounds containing protein nitrogen in the soil, and the hydrolysate is one of the nitrogen sources of plants (Manyun et al., 2018). In this study, PFA+SS treatment significantly increased the activities of S-UE, S-SC and S-NPT compared to SS treatment (**Table 1**), which might be attributed to the increased abundance of beneficial rhizosphere microorganisms. In our study, two-way ANOVA revealed highly significant positive PFA × SS treatment interactions on the enzyme activities (S-UE, S-SC, S-NPT) and physicochemical property (NN) of soil, indicating the better improvement of soil nutrient status with PFA under salt stress. Overall, PFA significantly improved the soil nutrient status under salt stress, such as NN, S-UE and S-NPT of rhizosphere soil.

Rhizosphere microbes play key roles in maintaining soil quality and affecting the host plant growth (Shang et al., 2021; Zheng et al., 2021; Sun et al., 2022). Compared to CK, PFA treatment decreased the bacterial Chao 1 index in the rhizosphere soil samples, suggesting that PFA affected the bacterial diversity, however, PFA had positive influence on the bacterial activity under salt stress (**Figure S3**). PCoA showed that PFA obviously shifted the rhizosphere microbial community composition of citrus (**Figure 4**). Qiao et al. (2022) also reported that the bacterial and fungal community were affected by potassium humate and PFA. Consequently, the rhizosphere microbial community affected by PFA was beneficial to the alleviation of salt damage to the seedlings.

The dominant bacterial phylum in the root and rhizosphere soil samples of the seedling was Proteobacteria (**Figures 5A, B**), which is easier to survive in the environments rich in multiple nutrients (Trivedi et al., 2013). The bacteria in Actinobacteria can enhance host nutrition acquisition and protect plant against various abiotic stresses (Shi et al., 2019), which was also found to widely exist in the rhizosphere soil of the seedlings (**Figure 5A**). At the genus level, PFA+SS treatment enhanced the relative abundances of *Bacillus* (**Figure 5C**), which had positive correlations with S-NPT, S-SC and S-UE, but negatively correlated with EC value (**Figure 6C**). *Bacillus*, a kind of rhizosphere beneficial bacteria, plays important roles in improvement of soil, promotion of plant growth, resistance against disease and enhancement of plant tolerance to salt (Miljaković et al., 2020; Shang et al, 2021). The changes of soil nutrient status can directly affect the morphological structure and function of rhizosphere microbial community, and the feedback effect also gradually affects the nutrient status and quality of soil (Sun et al., 2022; Yin et al., 2022). Therefore, the correlations between nutrient status and dominant microorganisms of the rhizosphere soil were evaluated in this study (**Figure 6**). These results suggested the evolutions of microbial community affected by PFA was closely related to the changes of soil nutrient status (OM, AN, NN, S-UE, S-SC and S-NPT), as supported by some studies (Zheng et al., 2021; Sun et al., 2022; Yin et al., 2022).

## Conclusion

PFA can effectively alleviate salt damage to citrus on yellowing and scorching of leaves. It showed obvious promotion effects on the physiological status of the seedlings and physicochemical properties and enzymatic activities of rhizosphere soil under salt stress. PFA improved the evolution of bacterial and fungal communities in root and rhizosphere soil of trifoliate seedlings, and significantly enhanced the relative abundances of *Bacillus*, a kind of rhizosphere beneficial microbes, under salt stress. Moreover, AP, S-SC and S-NPT affected by PFA were the most critical environmental factors influencing the compositions of microbial genera. Thus, PFA can be used as a potential conditioner to alleviate salt damage to citrus rootstocks.

## Data availability statement

The raw data has been deposited in the NCBI Sequence Read Archive under BioProject with the number PRJNA935538.

## Author contributions

MZ and SZ designed the experiments, analyzed the data and wrote the manuscript. MZ, XL, XW and JF performed the laboratory measurements. MZ and SZ discussed the results and provide critical idea in greenhouse experiment. All authors contributed to the article and approved the submitted version.
