# Supplementary material for: Potassium fulvic acid alleviates salt stress of citrus by regulating rhizosphere microbial community, osmotic substances and enzyme activities
Source: Front Plant Sci. 2023 Mar 23;14:1161469. doi: 10.3389/fpls.2023.1161469 (PMC10076529; doi:10.3389/fpls.2023.1161469)
Supplement: Supplementary file 1 [file DataSheet_1.docx]

**Supplementary Information**

**Lists of Supplemental Figures**

**Figure S1** Plant height (a), root length (b), fresh biomass (c), and dry biomass (d) of the seedlings.

**Figure S2** The Venn diagram of microbial community at the OTU level of different treatments.

**Figure S3** Alpha diversity indices of bacteria and fungi of different treatments.

**Figure S4** The relative abundance and composition of fungal communities in the rhizosphere soil (a) and root (b) samples at the phylum level. The significance comparisons of fungal communities in the rhizosphere soil (c) and root (d) samples at the genus level.


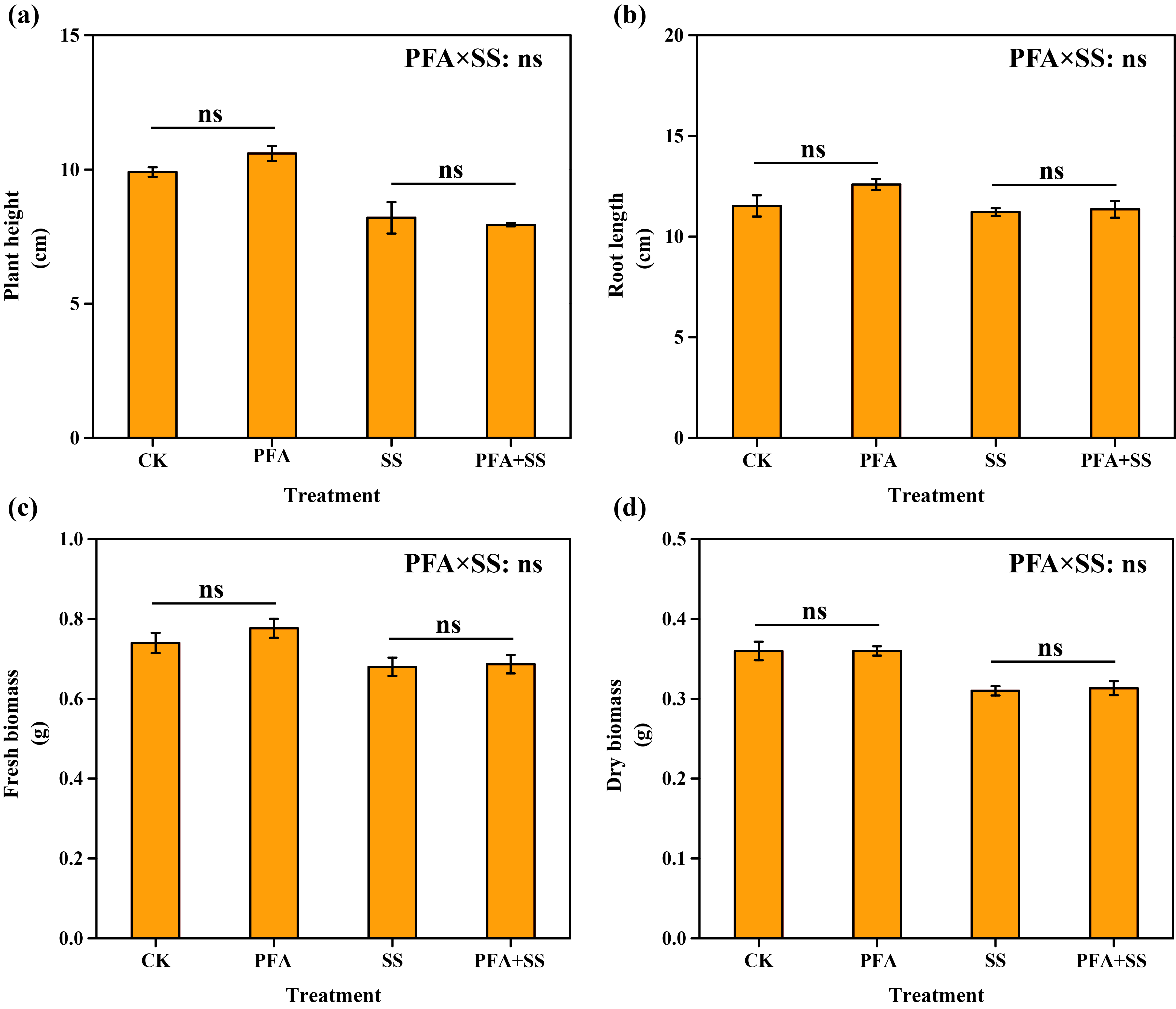


**Figure S1.** Plant height (a), root length (b), fresh biomass (c), and dry biomass (d) of the seedlings. CK: the seedlings without potassium fulvic acid and salt stress; PFA: the seedlings amended with potassium fulvic acid and without salt stress; SS: the seedlings with salt stress; PFA+SS: the seedlings amended with both potassium fulvic acid and salt stress. The error bars represent the standard error (n = 3). “ns”, “*”, and “**” mean not significant, *p* < 0.05 and *p* < 0.01 according to Student's t test and two-way ANOVA, respectively.


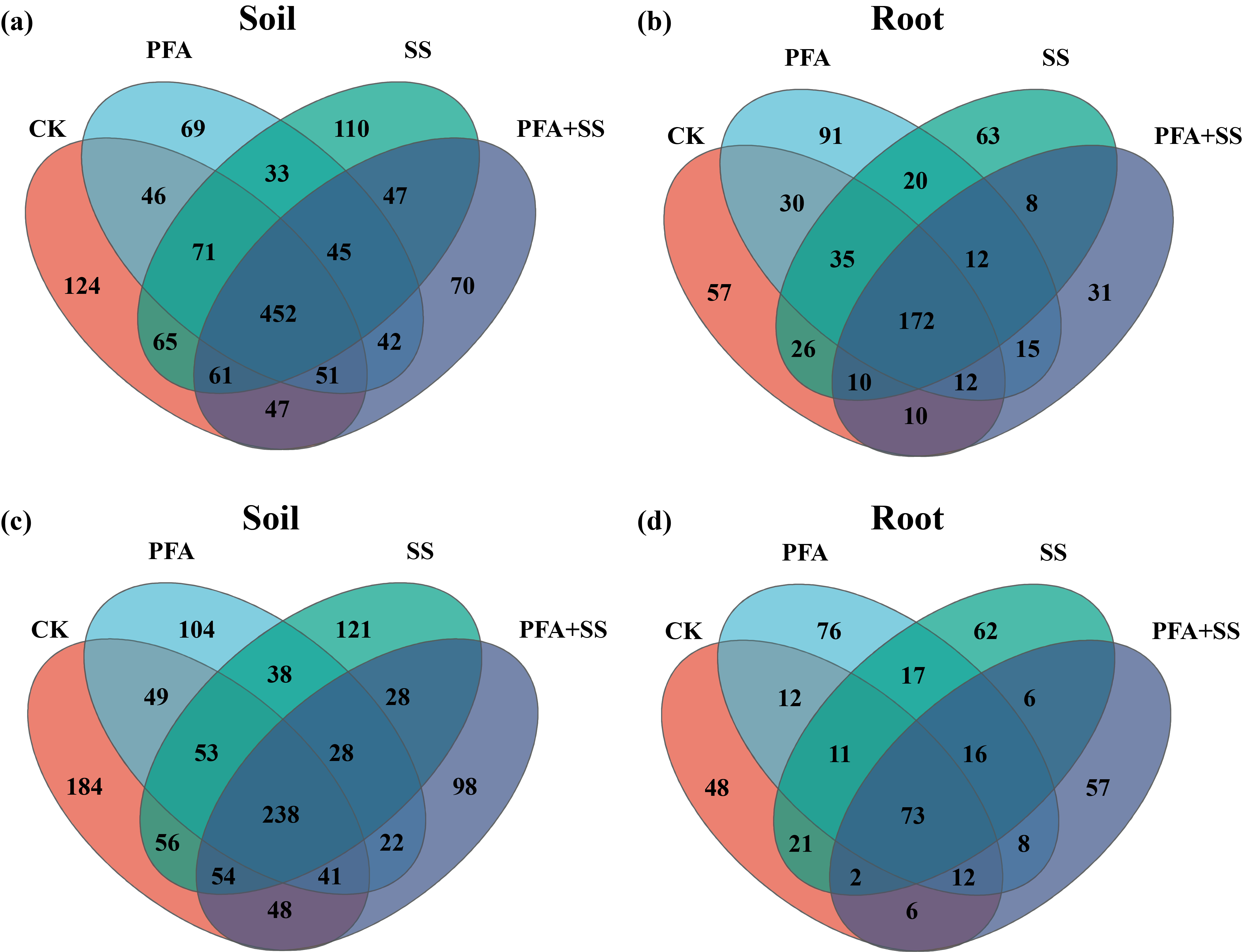


**Figure S2.** The Venn diagram of microbial community at the OTU level with different treatments. (a) Bacterial community for the rhizosphere soil sample; (b) bacterial community for the root sample; (c) fungal community for the rhizosphere soil sample; (d) fungal community for the root sample. CK: the seedlings without potassium fulvic acid and salt stress; PFA: the seedlings amended with potassium fulvic acid and without salt stress; SS: the seedlings with salt stress; PFA+SS: the seedlings amended with both potassium fulvic acid and salt stress.


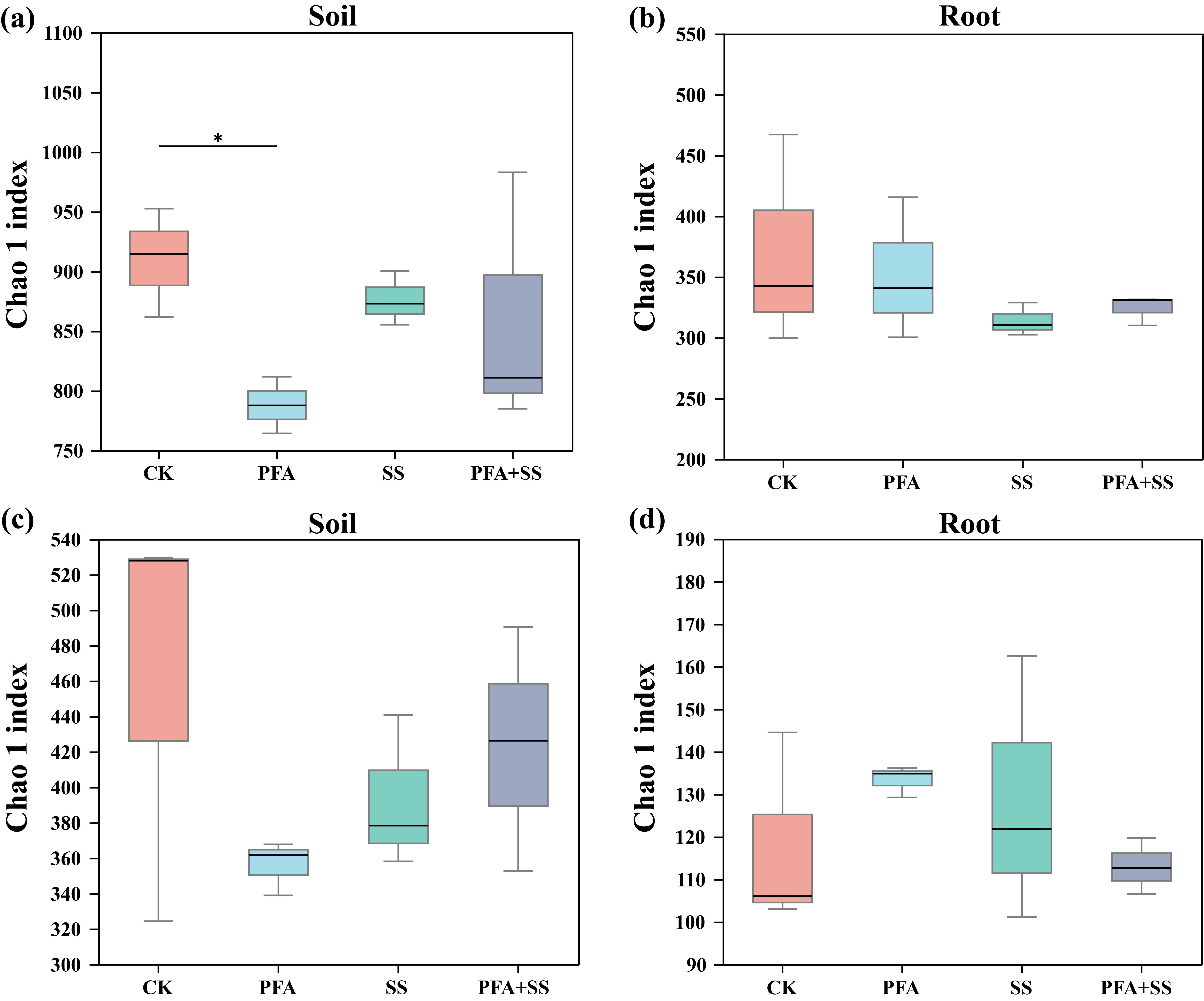


**Figure S3.** Alpha diversity indices of bacteria and fungi with different treatments. (a) Bacterial Chao1 index for the rhizosphere soil sample; (b) bacterial Chao1 index for the root sample; (c) fungal Chao1 index for the rhizosphere soil sample; (d) fungal Chao1 index for the root sample. CK: the seedlings without potassium fulvic acid and salt stress; PFA: the seedlings amended with potassium fulvic acid and without salt stress; SS: the seedlings with salt stress; PFA+SS: the seedlings amended with both potassium fulvic acid and salt stress. The asterisks (*) indicate significant difference among the treatments at *p*＜0.05.


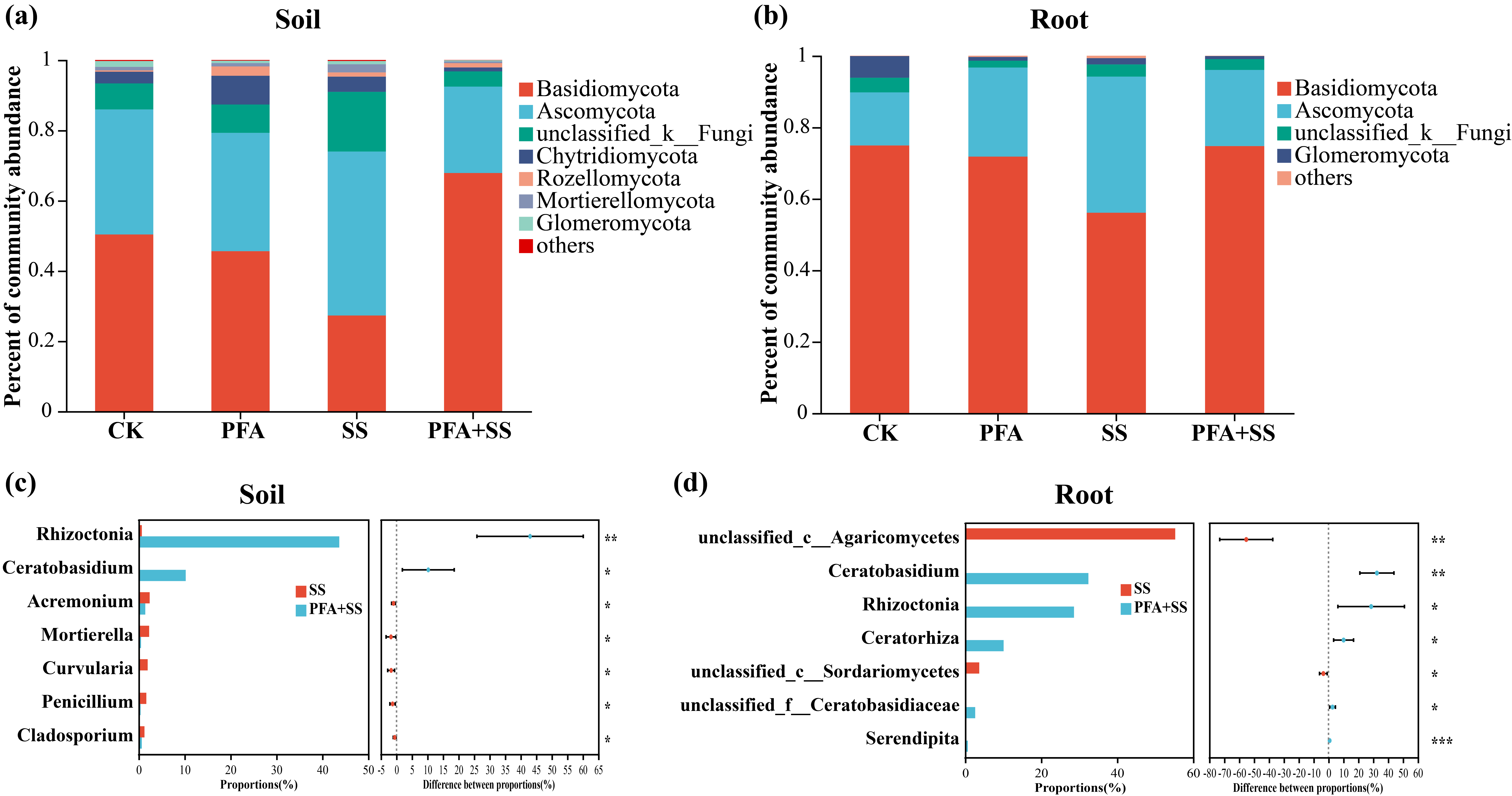


**Figure S4.** The relative abundance and composition of fungal communities in the rhizosphere soil (a) and root (b) samples at the phylum level, the significance comparisons of fungal communities in the rhizosphere soil (c) and root (d) samples at the genus level. CK: the seedlings without potassium fulvic acid and salt stress; PFA: the seedlings amended with potassium fulvic acid and without salt stress; SS: the seedlings with salt stress; PFA+SS: the seedlings amended with both potassium fulvic acid and salt stress. *** *p*＜0.001, ** *p*＜0.01, and * *p*＜0.05.
